# Important Roles of PI3K/AKT Signaling Pathway and Relevant Inhibitors in Prostate Cancer Progression

**DOI:** 10.1002/cam4.70354

**Published:** 2024-11-01

**Authors:** Rui Wang, Zhen Qu, Ying Lv, Lu Yao, Yang Qian, Xiangyu Zhang, Longquan Xiang

**Affiliations:** ^1^ Department of Clinical Medicine Jining Medical University Jining China; ^2^ Department of Pathology Jining First People's Hospital Jining China

**Keywords:** inhibitor, phosphatidyl inositol 3 kinase, PI3K/AKT signaling pathway, prostate cancer, protein kinase B

## Abstract

Prostate cancer (PCa) is an extremely common malignant tumor of the male genitourinary system, originating from the prostate gland epithelium. Male patients are prone to relapse after treatment, which seriously threatens their health. Phosphoinositide 3‐kinase (PI3K)/protein kinase B (PKB, also known as Akt) plays an important role in the growth, invasion, and metastasis of PCa. This review aimed to present an overview of the mechanism of action of the PI3K/AKT signaling pathway in PCa and discuss the application prospects of inhibitors of this pathway in treating PCa, providing a theoretical basis and reference for its clinical treatment targets.

## 1 Introduction

Prostate cancer (PCa) is a common malignant tumor in the male genitourinary system. The incidence of PCa increases with age and varies significantly in different regions. The incidence is relatively high in developed areas such as Europe and the United States, and the detection rate of PCa has significantly increased due to serological prostate‐specific antigen (PSA) screening [1]. It is the second leading cause of cancer death in men after lung cancer in Europe and the United States [2]. Some studies have indicated a positive correlation between age and the prevalence of PCa. Although the incidence was relatively low in China, it has increased recently due to the aging population [3, 4]. At the same time, the early detection of PCa because of the ongoing advancements in PCa diagnosis methods, leading to a further rise in its incidence.

The phosphoinositide 3‐kinase (PI3K)/protein kinase B (AKT) signaling pathway is involved in the proliferation, migration, and invasion of PCa cells. Hence, recent studies increasingly focus on this pathway as a potential therapeutic target. One study provided a comprehensive review of the activation of the PI3K/AKT signaling pathway, its role in the pathogenesis and progression of PCa, and the therapeutic strategies targeting this pathway for PCa treatment. It also discussed the latest research progress on the role of PI3K/AKT/mammalian target of rapamycin (mTOR) signaling pathway in cancer development and the inhibitors of molecular targets, especially PI3K and AKT inhibitors. We recommend that readers refer to Maria et al. [5], Antonino et al. [6], Jiarui et al. [7], Rosalin et al. [8], Carmen et al. [9], Mateusz et al. [10], Rajesh et al. [11], and Mehrdad et al. for further updates [12]. In addition, only PI3KI inhibitors were highlighted in this review. If you want to know more about other inhibitors of PI3KII, please refer to the study by Davide et al. [13].

## 2 Activation of PI3K/AKT Signaling Pathway

### 2.1 PI3K

PI3K primarily comprises the catalytic subunit p110 and the regulatory subunit p85. The catalytic subunit p110 has multiple subtypes, including p110α, p110β, p110γ, and p110δ. PI3K can be categorized into three subclasses, Class I, Class II, and Class III, based on the different subtypes of the catalytic subunit p110 and the varying substrate molecules [14]. Class I can be subdivided into IA and IB based on the distinct catalytic subunits. Also, PI3K has three subtypes, PI3Kα, PI3Kβ, and PI3Kδ [5], with p110α, p110β, and p110δ as the corresponding catalytic subunits, and *PIK3CA*, *PIK3CB*, *and PIK3CD* as the corresponding coding basis [6]. The *PIK3CA* gene, which encodes the catalytic subunit p110α, is strongly associated with the development of PCa [6]. Mutations in *PIK3CD*, the gene encoding P110δ, are rare and mainly associated with metastatic castration resistant prostate cancer (mCRPC) [15]. The regulatory subunits of IA consist of p85/p55, and the corresponding coding genes for the three subtypes are *PIK3R1*, *PIK3R2*, and *PIK3R3*. The upregulation of *PIK3R1* is closely associated with negative feedback in the androgen receptor (AR) signaling pathway and the PI3K signaling pathway during PCa development. Modulating p85 can render PI3KIA less active or inactive. Upon stimulation by external signals, the cells can activate PI3KIA through receptor tyrosine kinase (PTK), G protein‐coupled receptor (GPCR), rat sarcoma (RAS) or other ligands. This activation leads to the migration of PI3KIA to and binding on the inner side of the cell membrane, where it catalyzes phosphatidylinositol(4,5)bisphosphate (PIP2) conversion into phosphatidylinositol‐3,4,5‐trisphosphate (PIP3) [14]. IB has only one PI3Kγ, with p110γ as the corresponding catalytic subunit and *PIK3CG as* its coding gene [6]. The regulatory subunits include p101/p87, with *PIK3R5* and *PIK3R6* as their corresponding coding genes. However, genetic abnormalities in patients with PCa of this type are not common [14]. Class II is unique in that it consists of a single zymosome [14], unlike Class I and Class III, which are composed of heterodimers with catalytic and regulatory subunits. It can be further categorized into three isomers: PI3KC2α, PI3KC2β, and PI3KC2γ encoded by *PIK3C2A*, *PIK3C2B*, *and PIK3C2G*, respectively [7], all of which can catalyze the conversion of PIP2 into PIP3 [14]. The catalytic subunit VPS34 of Class III is encoded by *PIK3C3* [7], whereas the catalytic subunit VPS15 or p150 is encoded by *PIK3R4*. These genes are primarily associated with cell autophagy, and high levels of gene amplification of *PIK3R4* may contribute to the growth of PCa [14]. Classes I and II are the only ones capable of phosphorylating PIP2 to PIP3 [14].

### 2.2 AKT

AKT is a 57‐KDa serine/threonine protein kinase. The amino acid sequence in its active region is highly similar to protein kinase A (PKA) and protein kinase C (PKC). Therefore, AKT can also be referred to as protein kinase B (PKB) [14]. AKT consists of three subtypes: Akt‐α, Akt‐β, and Akt‐γ [5]. The structure primarily comprises three components: N‐terminal, C‐terminal, and middle region. The N‐terminal contains a plechstrinhomology (PH) domain that can bind to PI‐3,4,5‐P3 to facilitate AKT translocation to the cell membrane, leading to conformational changes and exposure of threonine at position 308 and serine phosphorylation sites at position 473. Simultaneously, phosphatidylinositol‐dependent protein kinase 1 (PDK1) binds to PI‐3,4,5‐P3 through the PH domain, bringing AKT and PDK1 close for threonine phosphorylation at AKT308. Subsequently, site 473 is phosphorylated under the influence of the mammalian target of rapamycin complex 2 (mTORC2), which activates all forms of Akt. The activated AKT then translocates from the cell membrane to the nucleus, where it binds with substrates to exert biological effects [14].

### 2.3 Both Upstream and Downstream Proteins

The kyoto encyclopedia of genes and genomes (KEGG) pathway analysis shows that the overall process of the PI3K/AKT signaling pathway can be described as follows: The cells receive ligands such as external growth factors, pathogen‐related molecular patterns, antigens, cytokines, extracellular matrix, chemoattractants, hormones, and neurotransmitters. These ligands act on corresponding receptors, such as receptor tyrosine kinases, Toll‐like receptors, B‐cell antigen receptors, cytokine receptors, integrins, and G‐protein‐coupled receptors, further activating downstream PI3K (only G‐protein‐coupled receptors activate Class 1A; others activate Class I B). Activated PI3K migrate to the inner membrane of the cell membrane to phosphorylate PI‐4,5‐P2 into PI‐3,4,5‐P3. The generated PI‐3,4,5‐P3 recruits PDK1 and AKT in cells and phosphorylates threonine at AKT308 to partially activate AKT [14]. Then, tryptophan at site 473 is phosphorylated under the action of mTORC2, and then all forms of AKT are activated [14]. The activated AKT is transferred to the cytoplasm [14]. PDK1 activates downstream ribosome S6 kinase (S6K1/2) and subsequently phosphorylates S6 to enhance protein synthesis [16]. Further, activate PKC is activated to enhance cellular glucose uptake [17]. The activation of protein kinase N (PKN) modulates actin recombination [18]. Also, the activation of serum and glucocorticoid‐induced kinase (SGK) promotes survival, growth, and proliferation of cells [19]. Therefore, the main function of PDK1 pathways is to promote cell proliferation. AKT triggers the activation of GTP hydrolases (GTPase). This activates the protein domain tuberous sclerosis complex (TSC1/2) and suppresses the small G protein (Rheb) from stimulating the mammalian target of the rapamycin (mTOR) complex in the mTOR signaling pathway, thereby facilitating protein synthesis [20, 21]. It stimulates endothelial nitric oxide synthase (eNOS) to generate nitric oxide (NO), leading to vasodilation, vascular restructuring, and angiogenesis [22]. It also activates breast cancer 1 gene (*BRCA1*) to promote DNA repair [23], murine double minute2 (MDM2) to degrade p53 through ubiquitination and promote cell growth [24], and ATP citrate lyase (ACLY) to promote fatty acid synthesis [25]. It inhibits glycogen synthase kinase 3β (GSK3) and activates glycogen synthase to promote glucose metabolism [26]. Furthermore, it inhibits the function of p21 and p27 to promote cell proliferation [27], forkhead box O (FOXO) to promote glycolysis and gluconeogenesis as well as cell proliferation and growth [28], and AS160 to promote glucose metabolism [29]. The PDK1 pathways involve three crucial proteins: phosphatase and tensin homolog (PTEN), which functions as a phosphatase and dephosphorylates PI‐3,4,5‐P3 to PI‐4,5‐P2 [6], protein phosphatase 2A (PP2A), and pleckstrin homology domain leucine‐rich repeat protein phosphatases (PHLPP) [30, 31]. The latter two possess phosphatase activity and thus can deactivate AKT and inhibit the PI3K/AKT signaling pathway [30, 31]. Shidong et al. found that lysine (K)–specific methyltransferase 2D (KMT2D) act as an upstream regulator of PI3K, promoting the expression of target genes leukemia inhibitory factor receptor (*LIFR*) and Kruppel‐like factor (*KLF4*). LIFR can combine with IL‐6 to promote the phosphorylation of PI3K. KLF4 can promote the metastasis and proliferation of PCa cells.

### 2.4 Modulation of the AR Signaling Pathway by the Negative Feedback

The AR pathway is another important pathway responsible for the development and progression of PCa. AR are expressed not only in primary PCa, but also metastatic prostatic adenocarcinoma, where they play a role in cell proliferation, migration, and invasion [32].

AR belongs to the nuclear receptor (NR) superfamily and functions as a ligand‐dependent nuclear transcription factor [33]. This gene is situated on the long arm of X chromosome 11–12 (Xq11‐12), comprising primarily eight exons. The protein encoded by this gene consists of four domains: the amino–terminal domain (NTD) encoded by exon 1, the DNA–binding domain (DBD) encoded by exons 2 and 3, the hinge region encoded by exon 4, and the ligand‐binding domain (LBD) encoded by exons 5–8 [34]. NTD accounts for the majority of AR, housing activation function 1 (AF1) in its main effect region. AF1 consists of two activation units: Tau‐1 (aa 100–370) and Tau‐5 (aa 360–485). Tau‐1 (aa 100–370) facilitates the internal dimerization of AR, particularly in a CAG repeat sequence within NTD, the length of which is inversely correlated with the risk of PCa [35]. DBD consists of two zinc finger structures. One of these is an α‐helical N‐terminal zinc finger encoded by exon 2 that can interact with the nucleotides of hormone response elements in DNA, and the other is a zinc finger encoded by exon 3 containing conserved D‐box motifs (ASRND) [35]. The main function is to regulate the binding of AR and androgen response elements (AREs) and promote the dimerization of AR [34]. The hinge region contains two distributor‐dependent nuclear localization signal (NLS) that facilitate the translocation of AR into the nucleus [34, 35]. LBD contains activator 2 (AF2), which facilitates the binding of androgens to AR [34], but this region is susceptible to point mutations [35].

**FIGURE 1:**
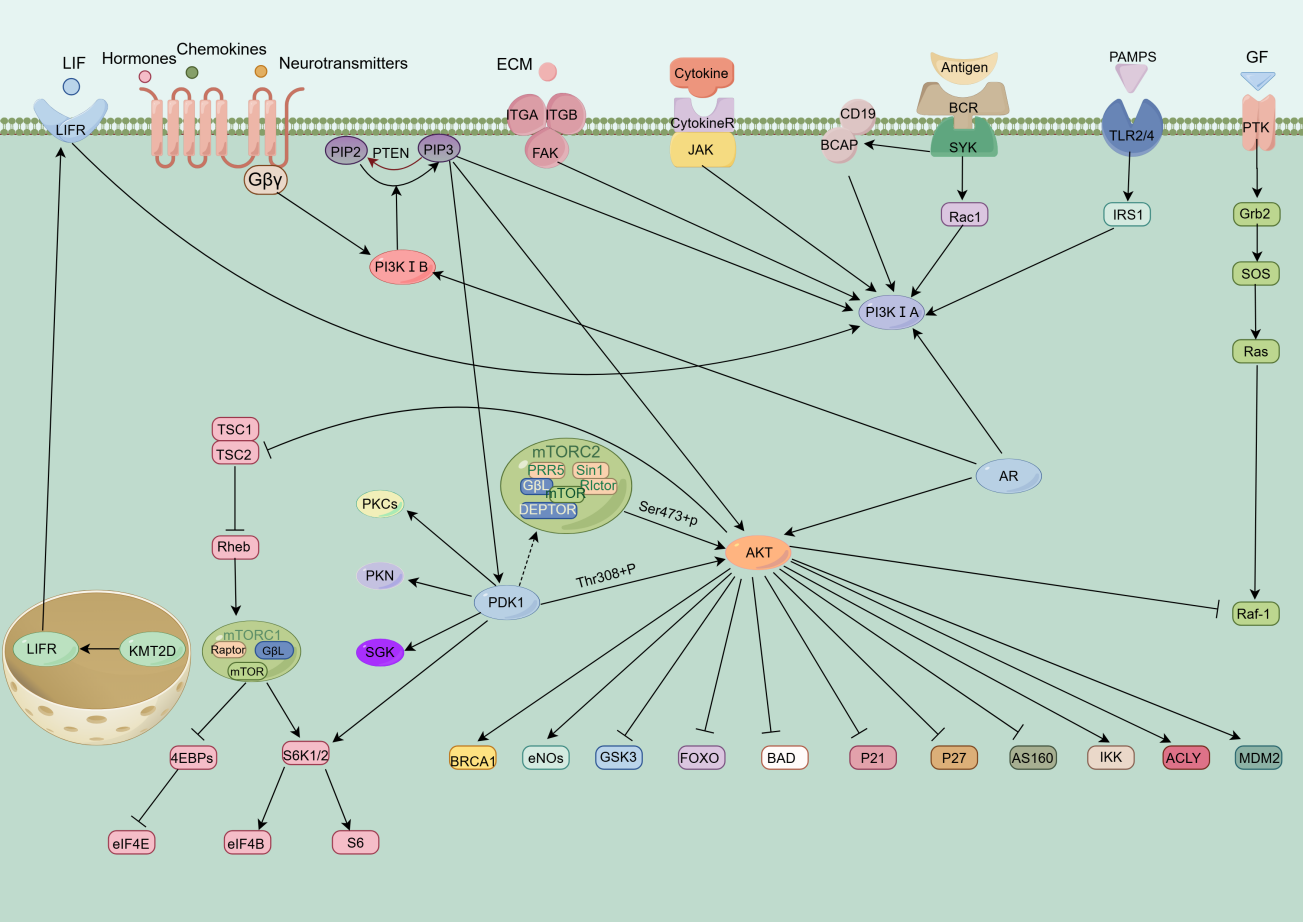
Schematic representation of the PI3K/AKT signal transduction pathway. Activation is indicated by arrows, and suppression is denoted by blocking lines.

The AR is typically situated in the cytoplasm, forming an inactive complex with heat shock protein 90 (HSP90) under normal conditions. Upon binding with androgens, AR disassociates from HSP90, undergoes internal dimerization, exposes its NLS, and subsequently translocates into the nucleus. The two AR molecules dimerize within the nucleus and bind to AREs to enhance the expression of target genes, particularly PSA [34], thereby facilitating the progression of PCa. Additionally, a negative correlation has been observed between the AR pathway and the PI3K/AKT pathway. Suppression of the AR pathway leads to decreased expression of FK506‐binding protein 5 (FKBP5) and inhibition of PHLPP function, thereby promoting AKT phosphorylation and activation [36]. Conversely, inhibiting the PI3K/AKT pathway allows the binding of an inhibitory agent with AR, promoting target gene expression [15]. A cross‐linking pathway exists between the PI3K/AKT pathway and the AR pathway in PCa development. Therefore, combination inhibitor therapy targeting both pathways should be prioritized to prevent drug resistance during treatment (Figure 1). For a detailed introduction to the impact of cross‐linking between AR and PI3K/AKT signaling pathway on PCa and its treatment, please refer to the studies by Sirin et al. [35], Boris et al. [36], and Fabio et al. [15].

## 3 Involvement of the PI3K/AKT Signaling Pathway in PCa Progression

The above description indicates that the PI3K/AKT signaling pathway primarily regulates anabolic processes and inhibits catabolism, promoting the survival of PCa cells and preventing apoptosis, and inducing the cell cycle process and proliferation rate of PCa cells to increase. In addition, some studies have found that activation of this pathway can promote epithelial–mesenchymal transition (EMT) and promote the metastasis of PCa cells [12]. So this signaling pathway is closely related to the development of PCa. Now, we will elaborate on the role of key molecules involved in the PI3K/AKT signaling pathway in PCa.

### 3.1 Impact of Upstream and Downstream Molecules on the Progression of PCa

S6K1/2 comprises three domains: the N‐terminal domain (NTD), the kinase domain (KD), and the carboxy‐terminal inhibitory domain (CTD). In the basal state, a connection exists between the C and N terminals. Upon phosphorylation of the C terminal, S6K1/2 undergoes a conformational change to an open state upon phosphorylation of the C‐terminal and can be activated by mTORC1 phosphorylation, thereby regulating transcription and facilitating protein synthesis [37].

PKCs are classified into three categories: conventional (c) PKCs, which include cPKCs‐α, βI, βII, and γ and require activation by Ca^2+^ or diacylglycerol (DAG); novel (n) PKCs, consisting of nPKCs‐δ, ε, η, and θ and requiring only DAG activation; and atypical (a) PKCs, containing aPKCs‐ζ, and ι(λ) and necessitating neither Ca^2+^ nor DAG activation [17]. PKC can collaborate with PKA to suppress the function of glycogen synthase, enhance glycogen metabolism, and facilitate glucose uptake by cells. The activation of nPKCs‐δ facilitates the migration of C‐X‐C motif chemokine ligand 8 (CXCL8) or C‐X‐C motif chemokine ligand 10 (CXCL10) to PCa cells [17].

PKN is a member of the PKC superfamily with functions similar to those of PKC, which can regulate the cytoskeleton of the actin after activation [18].

Serum/glucocorticoid regulated kinase (SGK) is a proto‐oncogene that facilitates the progression of PCa by phosphorylating Ser‐253 and Thr‐32 of FOXO3A, leading to the translocation of FOXO3A from the nucleus to the cytoplasm. This subsequently inhibits cell cycle arrest and apoptosis induced by FOXO3A [19].

BRCA1 associated protein‐1 (BAP1) is a protein encoded by *BRCA1* and containing a ring domain at the N‐terminal, which can form heterodimers with BRCA1 associated RING domain 1 (BARD1). Together, they function as a cell cycle progression factor to facilitate DNA synthesis and promote the proliferation of PCa cells during the transition from the G1 phase to the S phase [23].

As an E3 ubiquitin ligase, MDM2 can bind to P53 to promote ubiquitination degradation, thereby inhibiting the effect of the tumor suppressor genes effect on DNA and promoting the growth of PCa cells [24].

ACLY is involved in the tricarboxylic acid cycle to facilitate the synthesis of fatty acids and further lipid synthesis, providing cells membrane building blocks, signaling lipid molecules and post‐translational modifications of proteins for cancer cells proliferation [25].

FOXO is a member of the transcription factor family. The inhibition of the phosphorylation of FOXO leads to its translocation from the nucleus to the cytoplasm, where it downregulates the expression of its target genes, promotes glycolysis, suppresses tumor necrosis factor–related apoptosis–inducing ligand (TRAIL) expression, inhibits apoptosis in tumor cells, and facilitates tumor cell growth and proliferation [28].

The phosphorylation of AS160 leads to its inhibition, which, in turn, promotes the expression of glucose transporter type 4 (GLUT4), glucose metabolism, and cell growth [29].

## 4 Use of PI3K/AKT Inhibitors in Treating PCa

Molecular targeted therapies for cancer have garnered increasing attention in recent years. Castration‐resistant prostate cancer (CRPC) recurs and becomes fatal after 2–3 years of treatment with androgen deprivation therapy (ADT) targeting the AR signaling pathway [34]. We primarily investigated non‐AR pathways as potential targets, focusing on the PI3K/AKT signaling pathway, which is particularly important and meaningful. Six primary categories of inhibitors target the PI3K/AKT pathway: mTORC1 inhibitors, mTORC1/mTORC2 dual inhibitors, pan‐PI3K inhibitors, isoform‐specific PI3K inhibitors, PI3K/TORC1/2 dual inhibitors, and AKT inhibitors [34]. This study mainly explored the inhibitors related to PI3K and AKT.

### 4.1 PI3K Inhibitor

The clinically common PI3K inhibitors encompass four main categories: pan‐PI3K inhibitors, isoform‐specific PI3K inhibitors, PI3K isoform dual inhibitors [5], and PI3K dual‐target inhibitors [7].

#### 4.1.1 Pan‐PI3K Inhibitors

All subtypes of PI3K Class I share similar sequences in their ATP‐binding sites, which leads to the development of pan‐PI3K inhibitors [13]. Pan‐PI3K inhibitors can target all four catalytic subunits in Class IA and Class IB of PI3K (p110α, p110β, p110δ, and p110γ), demonstrating nonselectivity [34] and belonging to ATP‐competitive inhibitors [5].

The recently developed pan‐PI3K inhibitor, 5d, exhibits potent inhibitory activity against all four subtypes of PI3K [38]. It can further suppress the phosphorylation and activation of downstream proteins AKT and S6 ribosomal protein (S6RP) within the PI3K/AKT signaling pathway by inhibiting PI3K, thereby impeding the growth and proliferation of PCa cells while promoting apoptosis [38]. Although 5d is used for managing PCa, its clinical side effects remain unknown, necessitating further research.

Copanlisib, the only pan‐PI3K inhibitor sanctioned by the Food and Drug Administration (FDA) [8], has exhibited favorable clinical therapeutic efficacy in solid tumors with *PIK3CA* mutation and a reduced incidence of adverse effects compared with other pan‐PI3K inhibitors [9]. The efficacy of the AKT inhibitor is significantly higher than that of the PI3K inhibitor for patients with PCa with PTEN deletion. Despite being a pan‐PI3K inhibitor, copanlisib demonstrates superior inhibition of PI3Kα compared with the other three isoforms. Therefore, regardless of the PTEN status, copanlisib exerts a potent inhibitory effect on the proliferation of PCa cells [39].

The nonselective inhibitors of PI3K are linked to an increase in off‐target effects. They are typically not recommended due to the ubiquitous expression of PI3Kα and PI3kβ in all cells, as well as the predominant expression of PI3kδ and PI3kγ in leukocytes [6], Selective PI3K inhibitors have been developed to address the off‐target effects associated with pan‐PI3K inhibitors.

#### 4.1.2 Isoform‐Specific PI3K Inhibitors

The biggest difference between the isotype‐specific PI3K inhibitor and pan‐PI3K is that the former can selectively inhibit four catalytic subunits in Class IA and IB [5]. PI3Kδ and PI3Kγ are predominantly expressed in leukocytes and hence closely associated with immune system disorders and hematologic diseases [5], thus not being the primary focus of investigation. Our attention was directed toward inhibitors targeting PI3Kα and PI3Kβ.

##### 4.1.2.1 PI3Kα Inhibitors

The mutation of the *PIK3CA* gene is the most prevalent factor in the onset and progression of PCa [14], making p110α in PI3Kα a crucial target for targeted therapy against PCa.

The PI3Kα‐selective inhibitor F8 is a derivative of the pan‐PI3K inhibitor ZSTK‐474, with the introduction of benzoyl and benzoylhydrazyl groups. This modification has resulted in significant anti‐proliferative activity on the PCa PC‐3 cell line [40].

##### 4.1.2.2 PI3Kβ Inhibitors

As previously mentioned, a negative feedback regulation exists between the AR and PI3K/AKT pathways. Decreased AR expression can lead to the upregulation of PI3K, particularly p110β, thereby promoting the growth, proliferation, and metastasis of PCa [10]. PI3Kβ inhibitors are particularly suitable for the type of PCa associated with the AR pathway. Recent studies have demonstrated the significant role of PI3Kβ in activating the PI3K/AKT pathway in CRPC with PTEN deletion, indicating that targeting PI3kβ inhibitors is a crucial strategy for advanced de‐castration‐resistant PCa [41].

The 4h‐pyridine‐[1,2‐a] pyrimidine‐4‐one derivative TGX221 functions as a PI3Kβ inhibitor [5], leading to the inhibition of AKT phosphorylation and suppression of PCa cell proliferation [10].

GSK2636771 is a highly selective ATP‐competitive inhibitor of PI3Kβ [42] that significantly reduces the tumor size and decreases PSA levels in CRPC [41]. However, compared with PI3Kα, PI3Kβ is involved in fewer pathways, resulting in minimal side effects [41], making it a key research focus.

#### 4.1.3 PI3K Isoform Dual Inhibitors

Fabiana et al. discovered that the sole inhibition of PI3K can induce a dormant state in tumor cells without inducing cell death [43], indicating that single‐agent inhibition of PI3K is not a primary treatment for patients with PCa.

BAY‐1082439 exhibits dual inhibition of PI3Kα and PI3Kβ, downregulates P‐gp, and mitigates multidrug resistance in tumors. However, its limited in vivo solubility, short half‐life, and high clearance rate restrict its efficacy in vivo. In response to these limitations, the development of tumors can be inhibited by the PBDF system created [44].

#### 4.1.4 PI3K Dual‐Target Inhibitors

In dual‐target inhibition, inhibiting PI3K can simultaneously inhibit additional proteins, and the combination of the two is more effective in treating diseases [7]. Clinically, PI3K dual‐target inhibitors mainly include eight types: dual phosphoinositide 3‐kinase/histone deacetylases (PI3K/HDAC) inhibitors, dual PI3K/mTOR inhibitors, dual phosphoinositide 3‐kinase/poly ADP‐ribose polymerase 1 (PI3K/PARP) inhibitors, dual phosphoinositide 3‐kinase/bromodomain and extra terminal (PI3K/BET) inhibitors, dual phosphoinositide 3‐kinase/epidermal growth factor receptor (PI3K/EGFR) inhibitors, dual phosphoinositide 3‐kinase/heat shock protein 90 (PI3KHSP90) inhibitors, dual phosphoinositide 3‐kinase/mitogen‐activated extracellular signal‐regulated kinase (PI3K/MEK) inhibitors, and dual phosphoinositide 3‐kinase/extracellular regulated protein kinases (PI3K/ERK) inhibitors [7]. An overview of the first two types are presented here as they are more common.

##### 4.1.4.1 Dual PI3K/HDAC Inhibitors

Histone deacetylases (HDACs) are enzymes that can remove acetyl groups from histones, leading to a closer association between histones and inhibition of gene expression. The balance between histone deacetylases and histone acetyltransferases is normally dynamic, but when disrupted, it can contribute to cancer development [34]. Therefore, HDAC is a potential therapeutic target for PCa.

Recent studies have demonstrated that fimepinostat (CUDC‐907) is a newly developed multitarget drug designed for treating PCa. It is created by incorporating the HDAC inhibitory group hydroxamic acid into the core structure scaffold of the PI3K inhibitor [45], enabling it to effectively inhibit all three catalytic subunits in PI3KIA and HDAC. The combination of PI3K and HDAC inhibitors has shown significant antitumor activity, with relatively minimal side effects and a higher safety profile compared with PI3K and HDAC inhibitors individually [34].

LASSBio‐2208 is a novel dual inhibitor targeting both PI3K and HDAC6. It effectively suppresses the expression of janus kinase (JAK), leading to decreased levels of signal transducer and activator of transcription3 (STAT3) and interleukin‐ 6 (IL6) by inhibiting the PI3K/AKT pathway. Consequently, it inhibits the proliferation and metastasis of PCa [43].

Fimepinostat is also a dual inhibitor of PI3K/HDAC, which induces DNA damage and promotes apoptosis. The Notch pathway can also be activated to inhibit achaete‐scute family basic helix‐loop‐helix (BHLH) transcription factor 1 (ASCL1) expression for treating androgen receptor‐active prostate cancer (ARPC) and neuroendocrine prostate cancer (NEPC). The CRPC phenotypes include androgen receptor–active prostate cancer (ARPC) and neuroendocrine prostate cancer (NEPC) [46].

##### 4.1.4.2 Dual PI3K/mTOR Inhibitors

The mTOR complex exists in two primary forms: mTORC1 and mTORC2. mTORC1 promotes the growth and proliferation of PCa cells by enhancing the synthesis of proteins, lipids, and nucleotides through downstream molecules. Meanwhile, mTORC2 mainly activates downstream AKT to participate in the growth and proliferation of tumor cells [6]. Therefore, mTOR represents a promising therapeutic target for managing PCa. However, a negative feedback regulation exists between mTOR and multiple pathways. The mammalian target of rapamycin/S6 kinase1/phosphoinositide 3‐kinase (mTOR/S6K1/PI3K) signaling pathway can increase the expression of PI3K by negative feedback when simply inhibiting mTOR, thereby promoting the occurrence and development of PCa [47]. Hence, a combination of mTOR inhibitors along with other inhibitors, such as PI3K/mTOR dual inhibitors, is commonly used. PI3K and mTOR, both belonging to the phosphatidylinositol‐3 kinase‐related kinases (PIKK) family, share similar structures [5]. As a result, PI3K/mTOR inhibitors can target not only the four catalytic subunits of PI3K Class IA and IB but also two mTOR complexes, serving as ATP‐competitive inhibitors [34].

As a dual‐target inhibitor of PI3K/mTOR, 15a is demonstrates a significant reduction in tumor growth in vivo and also shows pronounced hepatotoxicity. Compound 42 was synthesized based on the structure of 15a to mitigate the adverse effects of 15a, resulting in reduced side effects through enhanced inhibition [48].

BEZ235, P1103, and BGT226 can inhibit PI3K and mTOR. Additionally, when combined with radiotherapy, the former two can effectively suppress the growth of PCa cells, weaken DNA damage repair function, and promote cell apoptosis [49]. Cellular experiments and clinical phase I trials have demonstrated a significant inhibitory effect of BGT226 on PCa cell proliferation in vivo [49].

(E)‐2,4‐difluoro‐N‐(2‐methoxy‐5‐(4‐(3‐(4‐methylpiperazin1‐yl)‐3‐oxoprop‐1‐en‐1‐yl)quinolin‐6‐yl)pyridin‐3‐ yl)benzenesulfonamide (8i) is a newly developed PI3K/mTOR dual inhibitor, derived from 4‐acrylamide quinoline by introducing 4‐acrylamide subunits at the C‐4 position of the quinoline core [47]. Xiaodong et al. demonstrated the ability of 8i to significantly decrease the expression of proteins in the PI3K/AKT pathway, including AKT, S6 and eukaryotic translation initiation factor 4E (eIF4E)‐binding protein 1 (4EBP1), thereby further inhibiting protein and nucleotide expression in PCa cells. Additionally, 8i exhibits a prolonged half‐life with stable metabolism in vivo, leading to a significant inhibition of the proliferation and growth of tumor cells [47].

### 4.2 AKT Inhibitors

AKT inhibitors can be categorized into three groups based on their distinct mechanisms of action: ATP‐competitive inhibitors, allosteric inhibitors, and irreversible inhibitors [5].

#### 4.2.1 ATP‐Competitive Inhibitors

These inhibitors can competitively bind with the ATP‐binding site on AKT and inhibit AKT phosphorylation [5].

Capivasertib (AZD5363) is the FDA‐approved ATP‐competitive AKT inhibitor, demonstrating significant efficacy in suppressing the proliferation of PCa cells, both as a monotherapy and in combination with other treatments [5].

Ipatasertib is a powerful and highly selective ATP‐competitive AKT inhibitor. Ipatasertib alone can improve the prognosis of mCRPC more inefficaciously than abiraterone‐combined therapy [9, 10].

A‐443654 is an indazopyridine inhibitor that reversibly binds to the ATP‐binding site on AKT, leading to the inhibition of glycogen synthesis kinase phosphorylation in PCa cells, promotion of glycogen synthesis, and suppression of PCa cells growth [11].

CCT128930 is a rare selective ATP‐competitive AKT inhibitor with high specificity for suppressing AKT2. This inhibitor exhibits significant antitumor effects, particularly in patients with PCa with PTEN deletion [11].

#### 4.2.2 Allosteric Inhibitors

These inhibitors primarily interact with the PH domain of AKT, leading to alterations in the protein structure at this location and preventing substrate binding [5], thereby inhibiting AKT activation. Compared with ATP‐competitive AKT inhibitors, allosteric inhibitors have higher specificity and fewer side effects [5].

Triciribine (TCN) is the initial nucleoside derivative AKT allosteric inhibitor. It remains inactive until it enters cells and undergoes phosphorylation by adenosine kinase to become active TCN‐P [5]. TCN promotes the apoptosis and death of PCa cells [5].

BAY1125976 exhibits potent anti‐proliferative activity in PCa as an AKT allosteric inhibitor [10].

**FIGURE 2:**
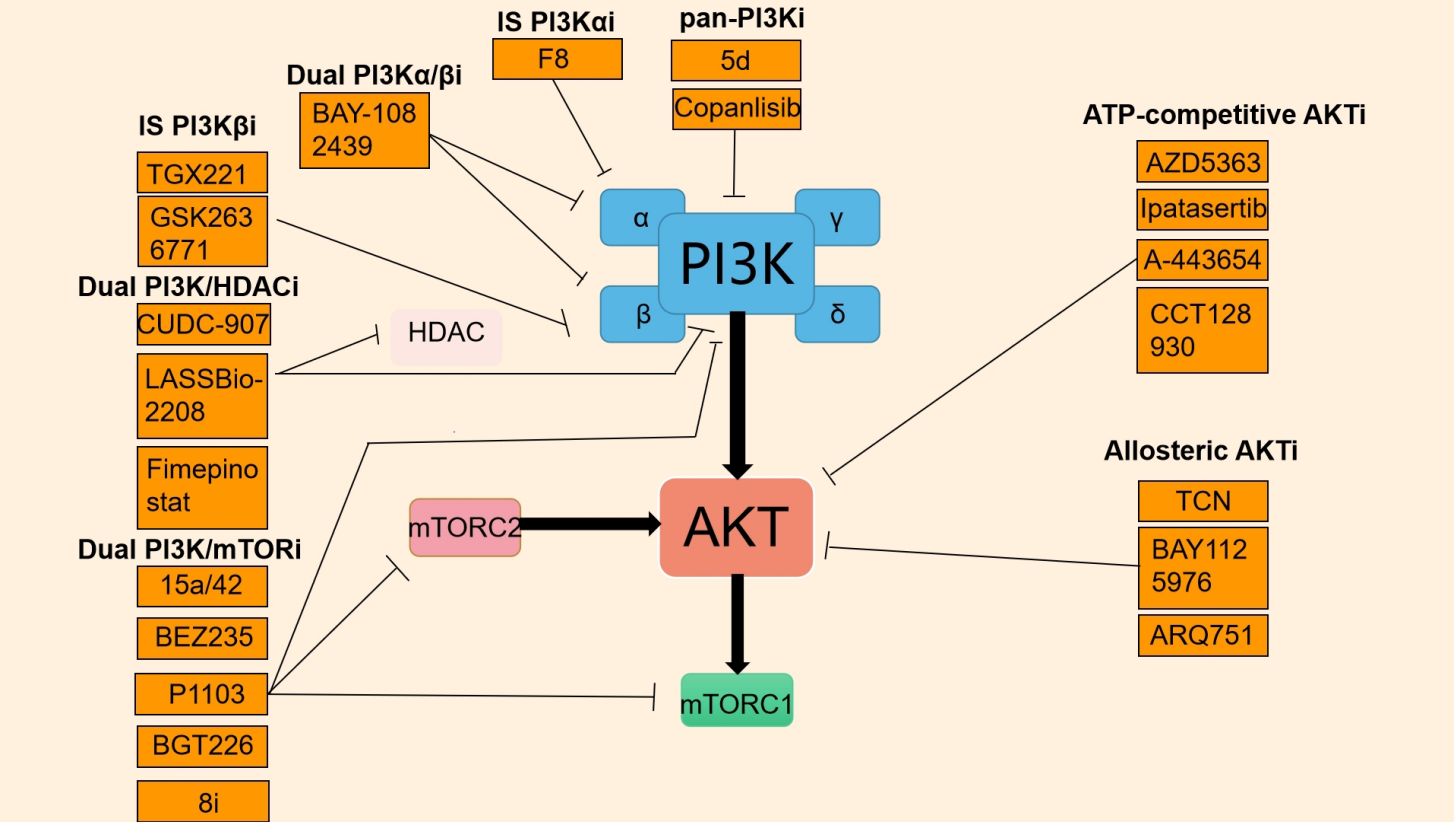
Targeted inhibition of PI3K, AKT, mTORC1/mTORC2, and HDAC. PI3K inhibitors encompass pan‐PI3K inhibitors (pan‐PI3Ki), isoform‐specific PI3K inhibitors (IS PI3Kαi, and IS PI3kβi), dual PI3K Isoform inhibitors (dual PI3Kα/βi), and PI3K dual‐target inhibitors (dual PI3K/HDACi and dual PI3K/mTORi). AKT inhibitors consist of ATP‐competitive AKTi and allosteric AKTi. Downstream activation of the PI3K/AKT pathway is indicated by arrows. The inhibitory effect of the inhibitor on the substrate is denoted by the blocking line.

Vevorisertib (ARQ751) is a highly selective allosteric AKT inhibitor that effectively suppresses AKT activation and pathway signaling, thereby impeding tumor progression. However, the PI3K/AKT signaling pathway is interconnected with various pathways. Despite the inhibition of this pathway, it may lead to the activation of compensatory pathways, resulting in vevorisertib resistance. Additionally, vevorisertib exhibits limited antitumor efficacy and is typically administered in combination with other drugs such as paclitaxel and fulvestrant. Nevertheless, studies have indicated that even when vevorisertib is combined with paclitaxel or fulvestrant, its antitumor effect remains predominantly moderate (Figure 2) [50].

### 4.3 Combined Drug Use

#### 4.3.1 AR Inhibitors Combined With PI3K/AKT Inhibitors

The negative feedback regulation between the AR and PI3K/AKT pathways has been identified [42]. When an AR inhibitor is used as a standalone treatment, it can transform PCa into CRPC [39]. This may eventually progress to mCRPC, resulting in the development of AR inhibitors resistance and ultimately leading to patient mortality [51]. Therefore, it is recommended to use AR inhibitors combined with PI3K/AKT pathway inhibitors. Studies have demonstrated that the dual‐target inhibitor of PI3K/mTOR samoolisib (LY3023414, GTPL8918) combined with the AR inhibitor enzalutamide significantly enhances the survival rates of patients with mCRPC [7]. Tatsuo et al. discovered that combining copanlisib, a pan‐PI3K inhibitor, and darolutamide, an AR inhibitor, synergistically enhanced DNA damage in PCa cells and promoted apoptosis [39]. Recent studies have demonstrated that the inhibition of AR protein expression using AR inhibitors enzalutamide, abiraterone, or antisense oligonucleotide (ASO) combined with AKT inhibitors AZD5363 and ipatasertib could effectively impede tumor cells progression [52, 53, 54]. Additionally, combining an AR inhibitor enzalutamide with a PI3Kβ inhibitor GSK2636771 has also shown similar efficacy [42]. However, Neal et al. demonstrated superior safety and efficacy of the combined use of AKT inhibitor capivasertib with abiraterone acetate (CYP17A1) in treating mCRPC compared with the combination of ipatasertib with abiraterone acetate [51]. Recagni et al. discovered that naphthalene diimide derivatives functioned as multitarget drugs capable of simultaneously inhibiting the AR pathway and acting as inhibitors of the PI3K/AKT pathway, thereby exerting antitumor effects [55].

#### 4.3.2 Other Drugs Combined With PI3K/AKT Inhibitors

Mohamed et al. discovered that the progression of PCa into advanced CRPC was primarily associated with two pathways: the PI3K/AKT/mTOR pathway and the hedgehog/glioma‐associated homolog (Hh/GLI) pathway. Within the Hh/GLI pathway, GLI plays a key role in driving the advancement of PCa. As a result, Mohamed et al. chose to combine dactolisib for PI3K/mTOR inhibition with GANT61 for GLI1 inhibition [56]. Dactolisib can promote the degradation of apoptosis protein D1 by activating glycogen synthesis kinase and reducing the activity of hypoxia‐inducible factor 1 (HIF‐1) to reduce the expression of vascular endothelial growth factor 1 (VEGF1). As the downstream molecules of GLI1, apoptotic proteins D1 and VEGF1 can be inhibited by GANT61 to reduce the expression of apoptotic proteins D1 and VEGF1 [56]. Thus, the coadministration of dactolisib and GANT61 can effectively suppress PCa progression by inducing apoptosis in PCa cells and inhibiting angiogenesis [56].

Gedatolisib (Ge) is a dual‐target inhibitor of PI3K/mTOR, and cabazitaxel is a second‐line chemotherapy drug for PCa. However, both have low selectivity and poor water solubility, and are highly toxic to patients. Therefore, disulfide cross‐linked micelles (DCM) are used to increase their water solubility and selectivity [57]. Nanoformulated gedatolisib (NanoGe) and nanoformulated cabazitaxel (NanoCa) were incorporated into DCM. When used in combination, they exhibited significant synergistic effects by co‐inhibiting the expression of anti‐apoptotic proteins B‐cell lymphoma‐2 (Bcl‐2) and cyclin D1, while increasing the expression of pro‐apoptotic protein Bcl‐2‐associated X‐protein (Bax). This led to a significantly enhanced efficacy against CRPC [57].

DS‐7324 is a dual inhibitor targeting PI3K/mTOR, which enhances the expression of metabotropic glutamate receptor subtype 1 (mGluR1) and human epidermal growth factor receptor 2 (HER2) in PCa cells with wild‐type PTEN PCa cells. This upregulation can lead to increased prostate‐specific membrane antigen (PSMA) expression by AR degradation, as well as elevated HER2 levels. PSMA levels also increase. Given that PSMA plays a role in tumor progression and drug resistance development, it may be beneficial to counteract this effect through combination therapy with mGluR1 or HER2 inhibitors [58].

Most cases of mCRPC are incurable. Docetaxel is the first‐line drug for mCRPC, but the mutation or deletion of PTEN can result in the development of docetaxel‐resistant mCRPC, making its treatment highly challenging [59]. To overcome resistance to docetaxel, the AKT inhibitor capivasertib, in combination with docetaxel, can effectively induce the apoptosis of PCa cells and cause DNA damage. This combination can potentially prolong overall survival (OS) but not progression‐free survival (PFS) [60, 61]. The synergistic antitumor effect of capivasertib is only observed after administering docetaxel. This is possibly due to cell cycle arrest in the G1/S phase following capivasertib treatment, which hinders the progression of docetaxel to the S/G2 phase and diminishes the antitumor efficacy of the combination [62]. Vicenc et al. discovered that resistance to docetaxel in mCRPC was associated with the activation of the PI3K/AKT and MEK/ERK pathways, as well as a negative feedback loop between these two pathways. Therefore, targeting both pathways simultaneously represents a promising therapeutic strategy. Vicenc et al. also found that combining MEK1/2 inhibitor selumetinib and PI3Kβ/δ inhibitor AZD8186 synergistically induced apoptosis in PCa cells and inhibited their proliferation [59]. David et al. found that AKT and PARP inhibitors alone could be used to treat mCRPC. Therefore, combining the two may be effective in treating mCRPC. In theory, the AKT inhibitor ipatasertib can induce tumor sensitivity to the PARP inhibitor rucaparib, and the combination of the two has potential synergistic effects. However, some individuals have withdrawn from the combination due to its adverse drug effects. Therefore, whether a synergistic relationship between ipatasertib and rucaparib exists for mCRPC remains unknown [63]. A previous study indicated that the combination of cyclin‐dependent kinase 4/6 (CDK4/6) inhibitors and AKT inhibitors had a synergistic effect in treating mCRPC [64]. CDK4/6 can activate cyclin D, phosphorylate retinoblastoma protein (Rb), and facilitate the cell cycle transition from the G1 phase to the S phase, thereby promoting tumor growth. The use of only a CDK4/6 inhibitor or an AKT inhibitor leads to drug resistance. On the one hand, the use of a CDK4/6 inhibitor alone stimulates the growth of PCa through the CDK4/6‐AKT axis, leading to resistance to the CDK4/6 inhibitor. On the other hand, using an AKT inhibitor alone can also increase the levels of phosphorylated proline‐rich Akt substrate of 40 kDa (pPRAS40) through alternative pathways, enhance P53 inactivation, promote cancer progression, and result in resistance to the AKT inhibitor. The combined use of both inhibitors can significantly decrease pPRAS40 levels, overcome resistance to CDK4/6 and AKT inhibitors, and synergistically contribute to anti‐mCRPC effects [64]. Sabina et al. highlighted the connection between provirus integration in Maloney murine leukemia virus (PIM) and PI3K pathways, as well as the potential for drug resistance with single drug use. In response, a study was conducted using a new multi‐kinase inhibitor AUM302, targeting PIM/PI3K/mTOR, along with PIM inhibitor AZD‐1208 and PI3K/mTOR inhibitor BEZ235. The results demonstrated that the synergistic combination of these inhibitors promoted cell apoptosis and inhibited cell proliferation. Furthermore, the combined use of PIM inhibitors with PI3K inhibitors exhibited superior efficacy compared with single‐targeted drug use, with AUM302 showing greater effectiveness than AZD‐1208 + BEZ235 [65].

Ezgi et al. discovered a crosstalk between the PI3K/AKT pathway and the Bcl‐2 pathway [66], indicating that Bcl‐2 is a downstream molecule of the PI3K/AKT pathway, thus providing a theoretical basis for the combined use of the two inhibitors. Erufosine (ErPC3) acts as an AKT inhibitor, inhibiting mTOR downstream activation by suppressing AKT, thereby facilitating cell apoptosis. Additionally, the Bcl‐2 inhibitor ABT‐737 can hinder the interaction between Bcl‐2 and Beclin‐1, removing the anti‐apoptotic protein Bcl‐2 and promoting apoptosis [66]. The combined use of erufosine (ErPC3), an AKT inhibitor, and ABT‐737, a Bcl‐2 inhibitor, exhibits a synergistic effect in promoting autophagy‐induced apoptosis in PCa cells [66].

The delivery of tumor necrosis factor‐related apoptosis‐inducing ligand (TRAIL) by mesenchymal stem cells (MSC) can stimulate the production of apoptosis‐inducing protease in PCa cells and facilitate apoptosis without causing harm to normal cells. However, its use may also enhance the expression of tumor cells transfer factor, although this side effect can be mitigated by using it in combination with an AKT inhibitor. Therefore, combining these two treatments shows potential for therapeutic application [67].

### 4.4 Other Treatments

As a tumor suppressor in PCa, switch defective (SWI)/sucrose non‐fermentable (SNF)‐ related, matrix‐associated, actin‐dependent regulator of chromatin subfamily c member 1 (SMARCC1) exhibits low expression levels, which can lead to the activation of the PI3K/AKT pathway, upregulation of cyclin D1/E1, and downregulation of cyclin‐dependent kinase inhibitor (CKI), thereby promoting PCa proliferation and facilitating EMT [68]. Restoring the expression of SMARCC1 can effectively suppress the PI3K/AKT pathway, thereby impede the progression of PCa [12].

Sirtuin 5 (SIRT5), which is the only type III histone deacetylase lowly expressed in PCa [69], is a tumor suppressor of PCa [12]. It can directly bind to PI3K to inhibit the PI3K/AKT signaling pathway, further inhibiting the nuclear factor kappa‐B (NF‐kB) pathway and reducing NF‐KB‐mediated angiogenesis and tumor invasion [69].

Protein‐L‐isoaspartate (D‐aspartate) O‐methyltransferase (PCMT1) is a widely distributed repair enzyme responsible for converting isomerized aspartic acid residues into their normal structures; it has been linked to PCa as an oncogene [70]. Low expression of PCMT1 can inhibit the activation of glycogen synthase kinase 3 beta (GSK3β), inhibit Snail expression, and promote the expression of E‐cadherin by inhibiting the PI3K/AKT pathway. Ultimately, this leads to the inhibition of EMT in PCa cells, thereby suppressing their invasion and metastasis [70].

Latent transforming growth factor beta‐binding protein 2 (LTBP2), a member of the extracellular matrix (ECM) glycoprotein superfamily, plays a regulatory role in the ECM and impacts tumor progression [71]. A recent study has demonstrated the ability of LTBP2 to recruit CD4+ T cells, suppress the PI3K/AKT pathway, and provide immunotherapy for PCa to impede its progression [71].

Resistance to paclitaxel typically develops in CRPC, but carbataxel has emerged as a new semi‐synthetic paclitaxel that can overcome this resistance. In a recent study, carbataxel inhibited the PI3K/AKT pathway, leading to an upregulation of Bax and caspase, as well as a downregulation of Bcl‐2. This combined effect ultimately promoted apoptosis and autophagy in PCa cells [72]. Meanwhile, carbataxel also increases the sensitivity of PCa to radiation therapy [72].

Fibroblast growth factor 21 (FGF21) is a member of the fibroblast growth factor (FGF) family. The liver primarily secretes it and plays a crucial role in metabolism. It has been closely linked to the progression of PCa [73]. FGF21 suppresses the proliferation of PCa cells and induces autophagy‐mediated apoptosis in these cells by inhibiting the PI3K/AKT/mTOR signaling pathway, thereby impeding the growth of PCa cells [73].

## 5 Summary and Prospects

Numerous studies have demonstrated a close association between the PI3K/AKT signaling pathway and the development of PCa, with its aberrant activation promoting tumor cell growth and proliferation. As a result, this pathway has garnered significant attention for potential treatment of PCa. PI3K and AKT inhibitors are used individually, but patients often develop resistance to these drugs. Furthermore, research has revealed a significant crosstalk between the PI3K/AKT pathway and numerous other pathways, with the most prominent being its interaction with the AR pathway. Therefore, the combined targeting of these two pathways represents a potential therapeutic approach for PCa. In addition, the discovery of some new drugs also contributes to reducing the side effects of drugs and improving the survival rate of patients. A large number of studies have shown that the PI3K/AKT signaling pathway is also cross‐linked with other signaling pathways, jointly promoting the occurrence and development of PCa. Therefore, exploring the mechanism of multiple pathways and cancer occurrence, finding crucial targets, and implementing multitarget combination drug use can provide new ideas and treatment strategies for the clinical treatment of PCa.

## Author Contributions

**Rui Wang:** conceptualization (lead), resources (supporting), software (supporting), writing – original draft (supporting), writing – review and editing (lead). **Zhen Qu:** resources (equal), software (equal), supervision (equal), writing – review and editing (equal). **Ying Lv:** resources (equal), supervision (equal), writing – review and editing (equal). **Lu Yao:** resources (equal), supervision (equal), writing – review and editing (equal). **Yang Qian:** resources (equal), supervision (equal), writing – review and editing (equal). **Xiangyu Zhang:** conceptualization (supporting), project administration (supporting), resources (supporting), software (supporting), supervision (supporting), writing – review and editing (supporting). **Longquan Xiang:** funding acquisition (supporting), project administration (supporting), resources (supporting), software (supporting), supervision (supporting), writing – review and editing (lead).

## Conflicts of Interest

The authors declare no conflicts of interest.

## Data Availability

Data sharing is not applicable to this article as no new data were created or analyzed in this study.
